# Translation and Validation of the Nomophobia Questionnaire in the Italian Language: Exploratory Factor Analysis

**DOI:** 10.2196/mhealth.9186

**Published:** 2018-01-22

**Authors:** Mohammad Adawi, Nicola Luigi Bragazzi, Lidia Argumosa-Villar, Joan Boada-Grau, Andreu Vigil-Colet, Caglar Yildirim, Giovanni Del Puente, Abdulla Watad

**Affiliations:** ^1^ Bar-Ilan Faculty of Medicine Padeh and Ziv Hospitals Zafat Israel; ^2^ Department of Health Sciences Postgraduate School of Public Health University of Genoa Genoa Italy; ^3^ Rovira i Virgili University Tarragona Spain; ^4^ Department of Computer Science State University of New York at Oswego Oswego, NY United States; ^5^ Sackler Faculty of Medicine Tel Aviv University Tel Aviv Israel; ^6^ Zabludowicz Center for Autoimmune Diseases Department of Medicine B Sheba Medical Center Tel Hashomer Israel

**Keywords:** nomophobia, questionnaire validation, exploratory factor analysis, psychometric properties

## Abstract

**Background:**

Nomophobia, which is a neologism derived from the combination of “no mobile,” “phone,” and “phobia” is considered to be a modern situational phobia and indicates a fear of feeling disconnected.

**Objective:**

No psychometric scales are available in Italian for investigating such a construct. We therefore planned a translation and validation study of the Nomophobia Questionnaire (NMP-Q), which is an instrument developed by Yildirim and Correia. Subjects were recruited via an online survey using a snowball approach.

**Methods:**

The NMP-Q was translated from English into Italian using a classical “backwards and forwards” procedure. In order to explore the underlying factor structure of the translated questionnaire, an exploratory factor analysis was carried out. A principal component analysis approach with varimax rotation was performed. Multivariate regression analyses were computed to shed light on the psychological predictors of nomophobia.

**Results:**

A sample of 403 subjects volunteered to take part in the study. The average age of participants was 27.91 years (standard deviation 8.63) and the sample was comprised of 160 males (160/403, 39.7%) and 243 females (243/403, 60.3%). Forty-five subjects spent less than 1 hour on their mobile phone per day (45/403, 11.2%), 94 spent between 1 and 2 hours (94/403, 23.3%), 69 spent between 2 and 3 hours (69/403, 17.1%), 58 spent between 3 and 4 hours (58/403, 14.4%), 48 spent between 4 and 5 hours (48/403, 11.9%), 29 spent between 5 and 7 hours (29/403, 7.2%), 36 spent between 7 and 9 hours (36/403, 8.9%), and 24 spent more than 10 hours (24/403, 6.0%). The eigenvalues and scree plot supported a 3-factorial nature of the translated questionnaire. The NMP-Q showed an overall Cronbach alpha coefficient of 0.95 (0.94, 0.89, and 0.88 for the three factors). The first factor explained up to 23.32% of the total variance, while the second and third factors explained up to 23.91% and 18.67% of the variance, respectively. The total NMP-Q score correlated with the number of hours spent on a mobile phone.

**Conclusions:**

The Italian version of the NMP-Q proved to be reliable.

## Introduction

New information and communication technologies (ICTs) have become particularly widespread and are increasingly utilized in modernized cultures. Due to their frequent use and their ubiquitous and ever-present nature, ICTs are often perceived as an irreplaceable part of a highly dynamic and interconnected society [1]. In particular, smartphones, as the latest evolution of mobile ICTs [2], have signaled the start of the *mobile age*. According to the 2015 Pew Research Center survey, in 2015 approximately two-thirds of Americans had a smartphone, and this figure is expected to grow in the coming years [3]. In Italy, according to the 2015 survey carried out by the National Institute of Statistics, 54% of families had a smartphone in 2014, which represents an upward trend with respect to 2013 [4].

However, although mobile devices enable users to perform a variety of tasks in an unprecedentedly rapid, easy, and effective way, they can also lead to serious medical problems. These problems include exposure to radiation, “screen dermatitis,” tumors, and infertility [5,6]; mobile devices can also interfere with driving safety and cause accidents [7,8]. Furthermore, the pervasiveness and intrusiveness of such devices could lead to mental health issues such as problematic use, distress, and compulsive usage, culminating in what is termed as “smartphone dependence” or “smartphone addiction” [9]. Some scholars consider this behavioral addiction to be a variant of technology addiction (or technopathy), while others consider it to be a specific addiction. Experts describe smartphone-related psychological disorders and syndromes, such as “short message service texting addiction,” compulsive selfie-taking behavior, *sexting*, or *phubbing*, among others [10]. *Sexting* can be defined as a behavior consisting of sending and receiving sexually explicit messages [11], whereas *phubbing* (snubbing someone in a social setting in favor of one’s own mobile phone) may lead to the impairment of social life and couple relationships [12].

Nomophobia, a neologism that is derived from the combination of “no mobile,” “phone,” and “phobia” has recently emerged as a modern problem, denoting the fear of feeling disconnected. Nomophobia is currently considered a situational phobia [13]. In more details, nomophobia can be characterized as:

The fear of not being able to use a smartphone or a mobile phone and/or the services it offers...the fear of not being able to communicate, losing the connectedness that smartphones allow, not being able to access information through smartphones, and giving up the convenience that smartphones provide [2]

Symptoms that characterize nomophobia include an excessive use of a mobile phone, which is kept permanently switched on, with the subsequent feeling of anxiety at the thought of a lack of network coverage. Other symptoms include the habit of continuously looking at the mobile screen in order to check for messages or missed calls (*ringxiety*, a combination of the words “ring” and “anxiety”), and the false sensation of hearing a mobile ring or vibration (the so-called “phantom vibration syndrome”) [13]. To the best of our knowledge, no psychometric scales are available in the Italian language for investigating this psychological construct. We therefore planned a translation and validation study of the Nomophobia Questionnaire (NMP-Q), which is an instrument developed by Yildirim and Correia [2].

## Methods

### Instrument

The NMP-Q was translated from English into Italian using a classical “backwards and forwards” procedure. The questionnaire was then administered, along with a general questionnaire comprising basic sociodemographic information (age, gender, schooling level) and average smartphone use.

### Study Design and Participants Selection

This investigation was designed as a cross-sectional study. Individuals were recruited via an online survey using a snowball approach that exploited Google Forms, which is an open-source tool for developing online questionnaires [14]. Given the exploratory nature of the study, a convenience sampling strategy was used. Online questionnaires were mainly circulated among undergraduate students and younger subjects, based on the idea that they were more likely to depend on their smartphones compared to other demographics. All procedures performed in the present study were in accordance with the ethical standards of the institutional research committee, and with the 1964 Helsinki declaration and its later amendments.

### Statistical Analysis

Continuous data were represented as means and standard deviations (SDs), while categorical data were expressed as percentages. Skewness and kurtosis were computed for each item score. Acceptable values for asymmetry/skewness and kurtosis are in the range from -2 to +2 in case of normal univariate data distribution. To investigate the factor structure of the translated questionnaire, an exploratory factor analysis (EFA) was carried out, using the principal component analysis (PCA) approach with *varimax* rotation performed on the 20 items of the questionnaire. *Varimax* rotation was chosen since it minimizes factor complexity while, at the same time, maximizing the variance of factor loadings [15]. Different PCA runs were conducted. First, an exploratory PCA was performed on the 20 items of the questionnaire without carrying out any rotation, in order to (1) check whether PCA could be deemed an appropriate technique for the matrix by examining if the correlations among items were >.30, and (2) control for the factorability of the correlation matrix using Bartlett’s test of sphericity. In cases of statistical significance, this test enables researchers to reject the null hypothesis (that is to say the correlations in the correlation matrix are zero and the matrix is an identity matrix).

The Kaiser-Meyer-Olkin (KMO) measure was calculated to assess the sampling adequacy. Ideally, the KMO should be >60. The likely number of factors was determined by (1) the number of factors with eigenvalues greater than 1 [15,16], and (2) a visual inspection of the scree plot. After checking the factor loadings, items were deleted in cases of unsatisfactory loading (values less than .45) or loading conflicting with a sound theoretical explanation. Different PCAs with *varimax* rotation runs were, therefore, carried out iteratively until a satisfactory, clearly interpretable solution was finally achieved. Cases of cross-loading were interpreted according to salience and explained variance, with theoretical considerations also being taken into account. Internal consistency and reliability was computed by calculating the Cronbach alpha coefficient for the scale and each found subscale. To interpret the alpha coefficient, the following rule of thumb was used [17,18]: excellent if >.9, good in the range .8-.9, acceptable in the range .7-.8, questionable or adequate in the range .6-.7, poor in the range .5-.6 and unacceptable if <.5. Multivariate regression analyses were computed to shed light on the psychological predictors of nomophobia. All statistical analyses were performed using the IBM Statistical Package for the Social Sciences (SPSS, version 22, NY, USA). Figures with *P* values less than .05 were considered statistically significant.

## Results

A total of 403 subjects (average age 27.91 years, SD 8.63; 160 males and 243 females, representing 39.7% and 60.3% of the sample, respectively) volunteered to take part in the study and were administered the Italian version of the NMP-Q (Textbox 1).

Regarding the schooling level, 2 subjects (2/403, 0.5%) had completed only elementary school, while 39 and 173 had completed middle school (39/403, 9.7%) and high school (173/403, 42.9%), respectively. All other subjects (189/403, 46.9%) had received higher education.

Concerning average smartphone use, 45 subjects usually spent less than 1 hour on their mobile phone (45/403, 11.2%), 94 spent between 1 and 2 hours (94/403, 23.3%), 69 spent between 2 and 3 hours (69/403, 17.1%), 58 spent between 3 and 4 hours (58/403, 14.4%), 48 spent between 4 and 5 hours (48/403, 11.9%), 29 spent between 5 and 7 hours (29/403, 7.2%), 36 spent between 7 and 9 hours (36/403, 8.9%) and 24 spent more than 10 hours (24/403, 6.0%). The means, SDs, and skewness and kurtosis figures are reported in Table 1. This information confirmed the normality of data distribution.

Since 403 subjects constituted a sample size large enough to compute reliable estimations of correlations among variables, we proceeded with the EFA. The N/k ratio (number of subjects/number of items) was 20.15:1, thus satisfying the requirements for commencing a factor analysis. Barlett’s test of sphericity was significant (Chi-square=5,796.275, degrees of freedom=19, *P*<.001) and the KMO index was .941.

**Italian version of the Nomophobia Questionnaire by Yildirim and Correia.**

1. Mi sento a disagio senza poter accedere costantemente alle informazioni tramite il mio smartphone

2. Sono infastidito/a se non riesco a cercare informazioni sul mio smartphone quando voglio farlo

3. Non essere in grado di ricevere le notizie (ad esempio gli ultimi aggiornamenti su eventi, meteo, ecc) sul mio smartphone mi rende nervoso/a

4. Sono seccato/a se non posso usare il mio smartphone e/o le sue applicazioni quando voglio farlo

5. L'idea di rimanere a corto di batteria nel mio smartphone mi spaventa

6. Se sono a corto di credito o se ho esaurito il mio limite di giga mensile, mi prende il panico

7. Se non c'è campo o non posso connettermi al Wi-Fi, rimango sempre a controllare per vedere se c'è segnale o se riesco a connettermi a una rete Wi-Fi

8. Se non posso usare il mio smartphone, ho paura di rimanere bloccato/a da qualche parte

9. Se non ho potuto controllare il mio smartphone per un po' di tempo, avverto il desiderio di controllarlo

Se non ho il mio smartphone con me,

10. Mi sento in ansia perché non riesco a comunicare istantaneamente con la mia famiglia e/o con gli amici

11. Sono preoccupato/a perché la mia famiglia e/o gli amici non possono raggiungermi

12. Mi sento nervoso/a perché non sono in grado di ricevere messaggi di testo e chiamate

13. Sono in ansia perché non riesco a rimanere in contatto con la mia famiglia e/o con gli amici

14. Sono nervoso/a perché non riesco a sapere se qualcuno mi ha cercato

15. Mi sento in ansia perché la mia connessione costante con la mia famiglia e gli amici è come se fosse rotta

16. Sono nervoso/a perché mi sento disconnesso/a dalla mia identità online

17. Sono a disagio perché non posso rimanere aggiornato/a con gli ultimi sviluppi dei social media e dei siti on-line

18. Mi sento a disagio perché non riesco a controllare le notifiche per gli aggiornamenti dei miei collegamenti e reti online

19. Mi sento in ansia perché non riesco a controllare i miei messaggi e-mail

20. Mi sento strano/a perché non saprei cosa fare

**Table 1:** Mean scores with standard deviation, skewness, and kurtosis.

Item Number	Mean	Standard deviation	Skewness	Kurtosis
1	3.610	1.720	0.122	–0.997
2	4.241	1.759	–0.263	–0.970
3	2.744	1.582	0.668	–0.426
4	3.945	1.775	–0.024	–1.019
5	3.663	1.790	0.179	–0.938
6	2.519	1.621	0.780	–0.484
7	2.732	1.694	0.938	0.063
8	2.390	1.631	1.126	0.428
9	3.975	1.793	–0.025	–0.972
10	3.591	1.835	0.132	–1.065
11	3.846	1.858	–0.024	–1.103
12	3.417	1.786	0.271	–1.016
13	3.588	1.825	0.172	–1.065
14	3.434	1.806	0.308	–0.944
15	2.675	1.716	0.762	–0.466
16	1.921	1.371	1.594	1.913
17	2.141	1.518	1.297	0.796
18	2.184	1.563	1.326	0.876
19	2.600	1.725	0.907	–0.189
20	2.285	1.609	1.158	0.406

As a result of the initial solution, four factors explaining up to 65.90% of the total variance were extracted. Before *varimax* rotation was performed, the first factor explained 23.32% of the variance, while the second and third explained 23.91% and 18.67%, respectively. After rotation, the three factors explained 23.32%, 23.91%, and 18.67% of the variance, respectively. The loadings of all items on each factor are shown in Table 2. Table 3 provides a summary of the EFA results and the reliability analysis of all items.

As seen in Table 2, the results of the second PCA run showed that each item loaded on a single factor, and that there was no cross-loading on other factors except for Item 5, which had a loading value of .452 on D1 and .520 on D3. This item was considered to load on its primary factor because this loading was more salient, thus explaining more variance, and was more theoretically reasonable. Furthermore, this value reached the cut-off value of .45; therefore, its loading on the secondary factor was not considered.

The Italian version of the NMP-Q showed an overall Cronbach alpha coefficient of .95 (.94, .89, and .88 for the three factors). As such, the internal consistency of the questionnaire can be considered good to excellent. The effect of dropping each variable per time is shown in Table 3. Removing each item per time leads to a decreased alpha coefficient for each item, showing that all items are essential to guarantee the questionnaire’s reliability.

The NMP-Q total score correlated with the number of hours spent using a mobile phone (standardized beta-coefficient=.385, *P*<.001), and with gender (borderline statistical significance: standardized beta-coefficient=.091, *P*=.057), whereas no statistically significant associations could be detected with age and schooling level (Table 4). Similar patterns were found for the total score of the NMP-Q subscale “not being able to access information,” with statistically significant associations with the number of hours spent on a smartphone (standardized beta-coefficient=.362, *P*<.001), gender (borderline statistical significance: standardized beta-coefficient=.088, *P*=.067) and schooling level (standardized beta-coefficient=.108, *P*=.024). The total score of the NMP-Q subscale “giving up convenience/losing connectedness” showed correlation only with the number of hours spent on a smartphone (standardized beta-coefficient=.402, *P*<.001). Finally, the score of the last NMP-Q subscale “not being able to communicate” correlated with the number of hours spent on a mobile device (standardized beta-coefficient=.254, *P*<.001) and with gender (standardized beta-coefficient=.142, *P*=.004).

In summary, the number of hours spent on a mobile phone turned out to be a predictor of all subscales, while gender was associated with D1 (although borderline significant) and D3. Finally, the schooling level correlated with D1. Further details are reported in Table 4.

**Table 2:** Factor loading of the Nomophobia Questionnaire. Salient factor loadings are indicated in italics.

Item Number	D1	D2	D3
1	0.255	0.307	*0.701*
2	0.235	0.110	*0.857*
3	0.201	0.301	*0.644*
4	0.257	0.186	*0.793*
5	0.452	0.292	*0.520*
6	0.273	*0.565*	0.368
7	0.143	*0.604*	0.359
8	0.366	*0.519*	0.105
9	0.334	0.394	*0.521*
10	*0.808*	0.255	0.244
11	*0.865*	0.078	0.255
12	*0.732*	0.299	0.356
13	*0.873*	0.159	0.272
14	*0.708*	0.369	0.328
15	*0.706*	0.438	0.137
16	0.265	*0.812*	0.108
17	0.163	*0.818*	0.198
18	0.156	*0.865*	0.194
19	0.235	*0.502*	0.253
20	0.136	*0.717*	0.208

**Table 3:** Cronbach alpha coefficient when one item is dropped.

Variable dropped	Raw alpha	Change in raw alpha	Standardized alpha	Change in standardized alpha
1	0.9420	-0.002916	0.9421	-0.002891
2	0.9428	-0.002099	0.9429	-0.002036
3	0.9431	-0.001823	0.9432	-0.001779
4	0.9424	-0.002526	0.9425	-0.002475
5	0.9417	-0.003142	0.9419	-0.003078
6	0.9422	-0.002689	0.9422	-0.002763
7	0.9432	-0.001728	0.9431	-0.001808
8	0.9440	-0.0009210	0.9440	-0.0009085
9	0.9419	-0.003009	0.9420	-0.002976
10	0.9409	-0.003969	0.9412	-0.003782
11	0.9422	-0.002717	0.9424	-0.002575
12	0.9402	-0.004725	0.9404	-0.004517
13	0.9410	-0.003918	0.9413	-0.003683
14	0.9399	-0.004983	0.9402	-0.004768
15	0.9412	-0.003729	0.9413	-0.003698
16	0.9423	-0.002553	0.9420	-0.002922
17	0.9423	-0.002538	0.9422	-0.002754
18	0.9420	-0.002886	0.9418	-0.003143
19	0.9443	-0.0006234	0.9443	-0.0006999
20	0.9434	-0.001453	0.9418	-0.003143

**Table 4:** Multivariate regression analysis investigating the impact of variables (age, gender, schooling level) on total and subscale scores.

Model		B	Standard deviation	Beta	*T*	*P* value
**Overall**	
	(Constant)	31.819	7.413		4.292	.000
	Number of hours spent using a mobile phone	4.450	0.590	.385	7.541	.000
	Age	0.105	0.141	.038	0.747	.455
	Gender	4.403	2.310	.091	1.906	.057
	Schooling level	0.801	1.352	.028	0.593	.554
**Not being able to access information**	
	(Constant)	10.722	2.556		4.195	.000
	Number of hours spent using a mobile phone	1.435	0.203	.362	7.055	.000
	Age	8.142E-005	0.049	.000	0.002	.999
	Gender	1.463	0.796	.088	1.837	.067
	Schooling level	1.052	0.466	.108	2.258	.024
**Giving up convenience/losing connectedness**		
	(Constant)	11.518	2.955		3.897	.000
	Number of hours spent using a mobile phone	1.849	0.235	.402	7.860	.000
	Age	0.044	0.056	.040	0.781	.436
	Gender	0.196	0.921	.010	0.213	.832
	Schooling level	-0.375	0.539	-.033	-0.697	.486
**Not being able to communicate**	
	(Constant)	9.579	3.056		3.135	.002
	Number of hours spent using a mobile phone	1.165	0.243	.254	4.790	.000
	Age	0.061	0.058	.056	1.057	.291
	Gender	2.745	0.952	.142	2.882	.004
	Schooling level	0.125	0.557	.011	0.223	.823

The impact on each item of age, gender, schooling level, and number of hours spent using a mobile phone is shown in Multimedia Appendix 1. Age had an effect on items 9 (borderline statistical significance: *P*=.063), 10 (borderline statistical significance: *P*=.089), and 15 (*P*=.003). The number of hours spent on one’s own mobile device affected almost all items (1-4, 6-10, 15-20), whilst for items 5, 11, and 12 a borderline statistical significance could be found, and items 13 and 14 had no significance. Gender impacted only items 8 and 11 in a statistically borderline way (*P*=.087 and *P*=.075, respectively). Finally, schooling level affected items 1 (*P*=.098), 2 (*P*=.074), 5 (*P*=.086), 15 (*P*=.085), and 19 (*P*=.086), whereas this parameter had a significant impact on items 9 (*P*=.021), 10 (*P*=.004), and 13 (*P*=.037).

The interaction between the number of hours spent on a mobile device and gender significantly affected only item 10 (*P*=.016), whereas this parameter resulted in statistically borderline results for items 2 (*P*=.095), 7 (*P*=.080), and 19 (*P*=.074). The interaction between the number of hours spent on one’s own smartphone and schooling level resulted in significance for items 9 (*P*=.003), 10 (*P*=.044), 11 (*P*=.019), and 18 (*P*=.010), and showed statistically borderline significance for items 2 (*P*=.073), 12 (*P*=.057), and 17 (*P*=.056). The interaction between schooling level and gender significantly impacted items 2-5. Finally, the interaction between the number of hours spent on a mobile phone, gender, and schooling level was statistically significant for items 3 (*P*=.011), 10 (*P*=.045), and 13 (*P*=.041), whereas they resulted in borderline significance for items 4 (*P*=.067), 16 (*P*=.066), and 20 (*P*=.053).

## Discussion

### Principal Findings

Based on the results of the reliability analysis, the internal consistency coefficient (Cronbach alpha) for the scale and all subscales of the NMP-Q was good, demonstrating that the NMP-Q is able to generate reliable scores. This finding is comparable with the alpha coefficient of the original instrument (Cronbach coefficient of .945, range of Cronbach coefficient for subscales from .819 to .939) [2]. The eigenvalues and scree plot supported a 3-factorial nature of the translated questionnaire.

These factors partially corresponded to the four factors found by Yildirim and Correia [2], namely items 1-5 and 9 (“not being able to access information”), items 6-8 (“giving up convenience”), items 16-20 (“losing connectedness”), and items 10-15 (“not being able to communicate”). The association between the scores and the number of hours spent using a mobile phone further corroborates and strengthens the validation of the NMP-Q in its Italian version. It is interesting to note that the gender effect using our instrument only had a statistically borderline influence. The few available studies and surveys that specifically assessed this point offer conflicting evidence [19,20]. This aspect warrants further investigations, dealing specifically with the gender issue.

The large sample size and the findings obtained in terms of psychometric properties represent the major strengths of our study. However, our research suffers from a number of limitations that should be properly recognized. First, since the sample is not representative, caution should be taken when making generalizations about our results. Further research should seek to replicate the results of the present study using more representative samples. Moreover, as for any other self-reported questionnaire, the self-reported structure of the NMP-Q may be a limitation due to social desirability bias. Nevertheless, there is a dearth of studies on nomophobia and this study makes it possible for researchers to use a reliable and validated instrument.

### Conclusions

Nomophobia is a modern, emerging, situational, mobile phone-related phobia. The Italian version of the NMP-Q was validated and its psychometric properties were examined, showing a 3-factor structure. The Italian NMP-Q proved to be reliable and can therefore be employed by researchers. Further studies are needed to assess the consistency of the NMP-Q in other samples (either general or clinical), and to investigate comorbidities and predictors of nomophobia using a confirmatory factorial approach to obtain more robust results. The relationship of nomophobia with other ICT-related psychological disorders (such as the Internet addiction) also warrants further investigations.

## Appendix

Multimedia Appendix 1 Impact of age, gender, number of hours spent using a mobile phone, and schooling level on items scores.

## Abbreviations

EFA: exploratory factor analysis
ICT: information and communication technology
KMO: Kaiser-Meyer-Olkin measure
NMP-Q: Nomophobia Questionnaire
PCA: principal component analysis
