# Supplementary material for: Translation and Validation of the Nomophobia Questionnaire in the Italian Language: Exploratory Factor Analysis
Source: JMIR Mhealth Uhealth. 2018 Jan 22;6(1):e24. doi: 10.2196/mhealth.9186 (PMC5799721; doi:10.2196/mhealth.9186)
Supplement: Multimedia Appendix 1 [file mhealth_v6i1e24_app1.pdf]

Multimedia Appendix 1

Variable	Item	F	Sig.
Age	1	.175	.676
	2	1.683	.195
	3	.808	.369
	4	.003	.954
	5	.062	.804
	6	.814	.368
	7	.034	.854
	8	2.031	.155
	9	3.483	.063
	10	2.905	.089
	11	.884	.348
	12	.545	.461
	13	1.730	.189
	14	.296	.587

	15	8.852	.003
	16	.320	.572
	17	.121	.728
	18	.299	.585
	19	.064	.801
	20	.294	.588
Number of hours spent using mobile phone	1	3.508	.001
	2	3.933	.000
	3	2.107	.042
	4	4.748	.000
	5	1.955	.061
	6	3.446	.001
	7	2.927	.005
	8	3.719	.001
	9	3.811	.001

	10	3.797	.001
	11	1.931	.064
	12	1.871	.073
	13	1.697	.109
	14	1.729	.101
	15	3.899	.000
	16	7.768	.000
	17	3.936	.000
	18	6.319	.000
	19	2.350	.024
	20	8.449	.000
Gender	1	1.724	.190
	2	.301	.583
	3	.003	.953
	4	.185	.668

5	1.118	.291
6	1.147	.285
7	.091	.763
8	2.944	.087
9	1.678	.196
10	2.452	.118
11	3.186	.075
12	.008	.930
13	1.303	.254
14	.055	.815
15	.643	.423
16	.483	.488
17	1.501	.221
18	.022	.883
19	2.510	.114

	20	.576	.449
Schooling level	1	1.975	.098
	2	2.149	.074
	3	.769	.546
	4	1.035	.389
	5	2.056	.086
	6	1.623	.168
	7	1.198	.312
	8	1.333	.257
	9	2.927	.021
	10	3.977	.004
	11	1.381	.240
	12	1.262	.285
	13	2.584	.037
	14	1.082	.365

	15	2.064	.085
	16	1.714	.146
	17	1.427	.224
	18	1.633	.165
	19	2.054	.086
	20	.545	.703
Number of hours spent using mobile phone * gender	1	1.244	.278
	2	1.756	.095
	3	1.579	.140
	4	1.200	.302
	5	.499	.835
	6	.202	.985
	7	1.832	.080
	8	.831	.562
	9	1.381	.212

	10	2.493	.016
	11	1.395	.206
	12	.619	.740
	13	1.687	.111
	14	1.080	.376
	15	1.099	.363
	16	1.200	.302
	17	.795	.592
	18	.416	.892
	19	1.869	.074
	20	.639	.724
Number of hours spent using mobile phone * schooling level	1	1.136	.310
	2	1.515	.073
	3	1.404	.117
	4	.661	.864

5	.839	.666
6	1.136	.310
7	1.027	.429
8	1.202	.250
9	2.138	.003
10	1.631	.044
11	1.805	.019
12	1.573	.057
13	1.442	.100
14	1.410	.114
15	1.161	.286
16	1.307	.171
17	1.578	.056
18	1.932	.010
19	1.341	.150

	20	1.132	.315
Gender * schooling level	1	2.031	.109
	2	3.179	.024
	3	5.821	.001
	4	3.337	.020
	5	4.341	.005
	6	1.921	.126
	7	.538	.657
	8	.786	.502
	9	.384	.765
	10	1.160	.325
	11	1.471	.222
	12	1.603	.188
	13	1.451	.228
	14	.139	.936

	15	1.043	.374
	16	.079	.971
	17	.641	.589
	18	.105	.957
	19	.185	.907
	20	2.089	.101
Number of hours spent using mobile phone * gender * schooling level	1	1.046	.407
	2	1.079	.373
	3	2.005	.011
	4	1.579	.067
	5	1.134	.319
	6	.871	.609
	7	.659	.843
	8	.983	.477
	9	.654	.847

10	1.679	.045
11	1.415	.127
12	1.359	.154
13	1.701	.041
14	.873	.606
15	1.407	.130
16	1.585	.066
17	.562	.919
18	1.047	.406
19	1.116	.336
20	1.639	.053
